# Research on Hybrid Feature Selection Method Based on Iterative Approximation Markov Blanket

**DOI:** 10.1155/2020/8308173

**Published:** 2020-04-07

**Authors:** Canyi Huang, Keding Li, Jianqiang Du, Bin Nie, Guoliang Xu, Wangping Xiong, Jigen Luo

**Affiliations:** ^1^School of Computer, Jiangxi University of Traditional Chinese Medicine, Nanchang 330004, China; ^2^School of Humanities, Jiangxi University of Traditional Chinese Medicine, Nanchang 330004, China; ^3^College of Pharmacy, Jiangxi University of Traditional Chinese Medicine, Nanchang 330004, China

## Abstract

The basic experimental data of traditional Chinese medicine are generally obtained by high-performance liquid chromatography and mass spectrometry. The data often show the characteristics of high dimensionality and few samples, and there are many irrelevant features and redundant features in the data, which bring challenges to the in-depth exploration of Chinese medicine material information. A hybrid feature selection method based on iterative approximate Markov blanket (CI_AMB) is proposed in the paper. The method uses the maximum information coefficient to measure the correlation between features and target variables and achieves the purpose of filtering irrelevant features according to the evaluation criteria, firstly. The iterative approximation Markov blanket strategy analyzes the redundancy between features and implements the elimination of redundant features and then selects an effective feature subset finally. Comparative experiments using traditional Chinese medicine material basic experimental data and UCI's multiple public datasets show that the new method has a better advantage to select a small number of highly explanatory features, compared with Lasso, XGBoost, and the classic approximate Markov blanket method.

## 1. Introduction

At present, due to the rapid development of scientific and technological level, the information acquisition technology and storage capacity have been greatly improved, and the data obtained carry more sufficient information, for which the scale is getting larger and larger. In the field of basic research of materials about traditional Chinese medicine, high-performance liquid phase (waters H-class) and mass spectrometry (synapt G2-si) are usually used to obtain experimental data. These data often involve thousands of substances, which are characterized by high-dimensional data and easily cause dimensional disasters. At the same time, due to the limitation of the experimental times, the characteristic of small samples is also presented, which easily leads to problems, such as overfitting. Conventional statistical analysis methods, such as multiple linear regression, principal component regression, and ridge regression, choose regression coefficients to reflect the relationship between variables [1–3], however, which cannot effectively delete irrelevant features and redundant features, and achieve the purpose of screening important substances for basic data of traditional Chinese medicines with high dimensionality and a small amount. At the same time, the traditional feature selection methods, such as Lasso and *K*-split Lasso [4], only can delete irrelevant features and redundant features to some extent and cannot meet the data processing requirements of high-dimensional small samples when dealing with data. Therefore, in view of the problem that high-dimensional small sample data of Chinese medicine contain more irrelevant information and redundant information, it is urgent to find an analytical model that can select effective features from high-dimensional small sample data, and improve the accuracy and operation of the model to provide technical support for researchers.

Next, this article will introduce the research-related work in Section 2. The new method is elaborated in Section 3. In Section 4, two basic data on TCM materials and three public UCI data are used to analyze in the new method, which is also compared with several existing algorithms to further verify the feasibility and effectiveness. Finally, the full text is summarized in Section 5.

## 2. Related Work

Feature selection is an effective method to solve dimensionality disasters and achieve feature dimensionality reduction. It can preserve the effective features that are most beneficial to regression (or classification) by analyzing the intrinsic relationship between features and target variables and features [5, 6], so that the redundant features and unrelated features to the target variable are better eliminated, aiming to reduce the complexity of the algorithm and improve the accuracy of the algorithm. According to the combination with machine learning, feature selection methods can be divided into filtering, encapsulation, embedded, and integrated [7]. Filtering is independent of a specific machine learning model, in which feature sorting and feature space search are generally used to obtain feature subsets including some special typical methods, for example, mutual information, symmetric uncertainty, and maximum information coefficient [8–10]. Encapsulation is to integrate the learning algorithm into the feature selection process, that is, the classification algorithm is regarded as a black box to evaluate the feature subset performance, which is to achieve the maximum classification accuracy rate. Embedded incorporates the feature selection process as the part into the learning algorithm. This method is used to solve the problem of high reconstruction cost when encapsulating different datasets. The integrated method is to gain the results, respectively, by learning using multiple feature selection methods firstly and then integrate each result with a certain rule. The method is better than the single feature selection method, which is suitable for solving the problem of instability of the feature selection method.

The feature selection method has attracted the attention of many domestic and foreign scholars. For example, in the field of biomedicine, Yao et al. [11] proposed a multimodal modal feature selection method based on hypergraph for multitask feature selection and finally selected effective brain region information; Sun et al. [12] proposed a hybrid feature selection algorithm based on Lasso, which can select a subset of information genes with strong classification ability; Mingquan et al. [13] proposed information gene selection method based on symmetry uncertainty and support vector machine (SVM) recursive feature elimination, which can effectively eliminate genes unrelated to categories. At the same time, feature selection methods are also well applied in other fields. Nagaraja [14] used partial least squares regression and optimized experimental design to select features with strong correlation with categories; Hu et al. [15] proposed feature selection algorithm by joint spectral clustering and neighborhood mutual information, which can remove signature-independent features.

However, the research methods mentioned in the above literature can only remove irrelevant features or eliminate redundant features to a certain extent and cannot meet the data processing needs of high-dimensional small sample problems of traditional Chinese medicine. Therefore, some researchers have conducted in-depth discussion and research to do a two-stage analysis of feature correlation and redundancy and approximated the approximate Markov blanket (AMB) to the feature selection process to achieve the purpose of screening effective and fewer features [16]. Among them, the literature [17] proposed a method of approximating the Markov blanket using cross entropy. The method first uses the Pearson coefficient to calculate the correlation between features and removes the irrelevant features and then uses the approximate Markov blanket to perform redundant features: deletion; the paper [18] proposed a maximum correlation minimum redundancy feature selection algorithm using approximate Markov blankets. The method first uses the criterion of maximum correlation minimum redundancy for feature correlation ordering and then does approximate calculation by combining mutual information with Marco to remove irrelevant features and redundant features; the literature [19] proposed a feature selection method based on the maximum information coefficient and approximate Markov blanket (FCBF-MIC), which firstly measured correlation between features and categories by symmetric uncertainty to delete the features that are not related to categories or weakly correlated. Secondly, the Markov carpet is approximated by using the maximum information coefficient, thereby achieving the purpose to delete redundant features. However, after the analysis and discussion of the experiment, it is found that the above method is more strict because of the definition of the approximate Markov blanket, which makes it impossible to select a small number of highly explanatory features in the high-dimensional small sample data of Chinese medicine, so it is still needed for us to do further research and exploration of Chinese medicine data analysis methods.

In a feature selection study, higher-quality feature selection methods should exhibit the following characteristics [20]: (1) interpretability, meaning that the features selected in the model have scientific significance; (2) acceptable model stability; (3) avoidance of deviations in the hypothesis test; and (4) model calculation complexity within a manageable range. At the same time, in the literature [21], a standard of optimal feature subsets is proposed into four categories: unrelated features, weakly correlated and redundant features, weakly correlated nonredundant features, and strongly correlated features. It is considered that the optimal feature subset should contain the latter two in this paper. Through a large number of experimental comparisons, the standard has been proved to have lower time complexity and better feature selection results [22, 23].

In view of this, this paper proposes a hybrid feature selection method based on iterative approximate Markov blanket (CI_AMB), which is divided into two phases: in the first phase, it first uses the maximum information coefficient to measure correlation between the per-dimensional features and target variables and achieves the filtering of unrelated features and the acquisition of candidate feature subsets according to some evaluation criteria; in the second stage, the candidate feature subsets are sorted and classified into *K* subsets and then iteratively cull redundant features to obtain weakly correlated nonredundant features and strongly correlated features based on the maximum approximate Markov carpet of information coefficients. Not only can the algorithm effectively filter the irrelevant features and eliminate redundant features, but also reduces the time complexity of the model and improves the interpretation degree of the model. It is a new model suitable for high-dimensional small sample data analysis of traditional Chinese medicine.

## 3. Research on Hybrid Feature Selection Method Based on Iterative Approximation Markov Blanket (CI_AMB)

The maximum information coefficient (MIC) is a new information-based metric proposed by Reshef et al. [24] in 2011. It not only better reflects the correlation between features and target variables, and features and features, but also makes up for the problem that metrics such as mutual information cannot be normalized and sensitive to discretization and that metrics such as information gain and symmetry uncertainty cannot effectively measure the nonfunction dependence between features. In many experimental analyses, the characteristic that the largest information coefficient has good stability and the ability to metric the relationship among the features are also effectively demonstrated [25–27].

The Markov blanket is a method that minimizes subset of features to keep maximizing the target variable information and meanwhile makes the remaining feature subset to be independent of the target variable under the conditions that subset of features has been selected [19, 28]. Although the Markov carpet can achieve the effect of feature dimension reduction, because its independent conditions are too strict and the relationships discovered belong to the NP-hard problem, the feature selection method often adopts the strategy of approximating the Markov blanket. Therefore, combining the advantages of the largest information coefficient, in this paper, we use MIC to approximate the Markov blanket (see Definition 1) in order to better eliminate the redundant features, so that the optimal feature subset screening and model optimization are achieved.

**Definition 1 .**

(approximate Markov blanket). Assume that there are two different features in the feature set, respectively, if

(1) $$\begin{array}{c} \text{MIC}\left(f_{i},\text{obj}\right)\geq \text{MIC}\left(f_{j},\text{obj}\right)\left\|\text{MIC}\left(f_{i},f_{j}\right)\geq \text{MIC}\left(f_{j},\text{obj}\right).\right. \end{array}$$

It is considered that *f*_*i*_ is an approximate Markov blanket of *f*_*j*_, that is to say, *f*_*i*_ is retained while *f*_*j*_ is a redundant feature and removed from the feature set.

**Definition 2 .**

(weakly correlated nonredundant features and strongly correlated features). Only when satisfying the condition that there is no an approximate Markov blanket to feature *f*_*i*_, the feature *f*_*i*_ is a weakly correlated nonredundant feature or a strongly correlated feature, namely, *f*_*i*_ ∈ {*F* − *f*_irrelevant_ − *f*_redundant_}, where *F* is the feature complete set and *f*_irrelevant_ and *f*_redundant_ are the irrelevant feature set and the redundant feature set, respectively.

**Figure 1:**
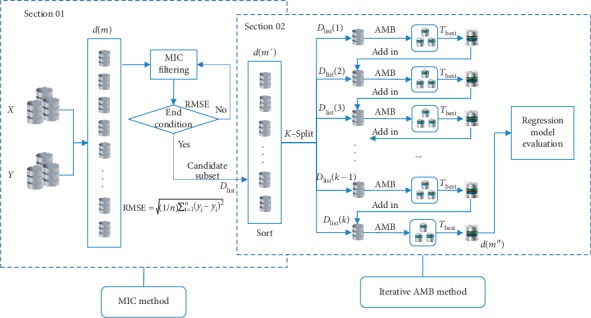
CI_AMB model.

The CI_AMB method is mainly divided into two stages. In the first stage, it firstly uses the MIC method to measure the correlation between each feature and the target variable and achieves the filtering of better irrelevant features according to the evaluation criteria to achieve the acquisition of candidate feature subsets. The features selected by the MIC method are usually highly correlated with the redundant features accompanied, in which the more amounts of the redundant features not only increase the time complexity and space complexity of the model, but also reduce the degree of interpretation of the model. Therefore, in the second stage, the new method further analyzes the redundancy of the feature, that is, according to the feature score obtained by the MIC method, the features of the candidate subset are arranged in ascending order and equally divided into *K* parts. And then, the approximate Markov blanket (AMB) is used to iteratively eliminate redundant features, so that weakly correlated nonredundant features and strongly correlated features can be selected (Algorithm 1). The flow of the algorithm construction is shown in Figure 1.

The specific construction process of the model is as follows:

 
*Phase 1*. Filtering irrelevant features
 
*Step 1*. MIC calculation: MIC calculation is performed on the original data with *m* features, that is, the maximum information coefficient is calculated for each feature by formula (2) and obtains a score sequence *T*_list_ = (*t*_1_, *t*_2_,…, *t*_*m*_) of all features, and the *T*_list_ value is [0, 1]. It is worth noting that the closer the score of the feature is to 1, the stronger the correlation of feature and the target variable is, and the closer the score is to 0, the weaker the correlation is:

(2) $$\begin{array}{c} \text{MIC}\left(O_{s}\right)=\underset{xy<B\left(n\right)}{\max}\frac{{\text{MI}}^{*}\left(O_{s},x,y\right)}{\log\min\left\{x,y\right\}}, \end{array}$$

  where MI^*∗*^(*O*_*s*_, *x*, *y*) refers to the maximum mutual information of *O*_*s*_ under mesh partitioning [19, 29], *O*_*s*_ is the ordered pair set of samples, *x* means dividing the value range of feature *X* into *x* segments, *y* means dividing the value range of dependent variable *Y* into *y* segments, and *B*(*n*) is the upper limit of the meshing. Generally, the value of *B*(*n*) is *B*(*n*) = *n*^0.6^, and *n* is the sample size.
 
*Step 2*. Determining the candidate feature subset: MIC calculation is used to obtain the score sequence *T*_list_, and the descending order is arranged and the sequence *T*_list_ is intercepted according to a certain ratio, and then the current top ranked feature subset is selected; if the selected feature subset satisfies the best of evaluation index RMSE, the candidate feature subset *D*_*m*′_ (*m*′ dimension feature, *m*′ < *m*) can be directly selected, but if not, the progress of filtering operation and judgment is continued:

(3) $$\begin{array}{c} \text{RMSE}=\sqrt{\frac{1}{n}\sum_{i=1}^{n}{\left(y_{i}-y_{i}\right)}^{2}}. \end{array}$$

 
*Step 3*. Data division and initialization: the candidate feature subsets *D*_*m*′_ are arranged in reverse order according to the feature scores, thereby obtaining an aligned candidate feature subset *D*_list_ in order to ensure the maximum retention for the features with high important correlation in the regression tasks by ranking the features in the later processing, then subdividing the candidate feature subset *D*_list_ into *K* groups, and defining *D*_list_(*i*) is the *i* − *th*(1 ≤ *i* ≤ *K*) feature after dividing the candidate feature subset into *K* groups subset, while initializing the optimal feature set *T*_best_ to be empty.
 
*Phase 2*. Eliminating redundant features
 
*Step 4*. Feature redundancy analysis: first, the redundant features are removed from the first one feature subset *D*_list_(1) by using the AMB method (i.e., Definition 1), and then the nonredundant features are filtered into *T*_best_. Secondly, *T*_best_ and the second one feature subset *D*_list_(2) are merged as the current feature subset, and it will be analysed by the AMB method to delete the redundant features, and then the *T*_best_ is updated currently. Therefore, the optimal feature subset *T*_best_ with the remaining the m″ dimension (*m*″<*m*′) is obtained by iterating sequentially to the k-th feature subset *D*_list_(*k*) in order.
 
*Step 5*. Model evaluation: compare and evaluate various strategies by using the weakly correlated nonredundant and strongly correlated optimal feature subsets (*T*_best_) obtained in the above steps.

## 4. Experimental Design

### 4.1. Experimental Data Description

The five experimental datasets were used in this paper including the traditional Chinese medicine material basic experimental data (WYHXB and NYWZ) of the Modern Chinese Medicine Preparation Ministry of Education, the Residential Building Dataset (RBuild), Communities and Crime on the UCI dataset (CCrime), and BlogFeedback (BlogData for short), and the basic information of each dataset is described in Table 1. Among them, there are 798 features, 1 dependent variable, and 54 samples in WYHXB data, and 10283 features, 1 dependent variable, and 54 samples in NYWZ data; BlogData is data describing blog posts, which includes 280 features, 1 dependent variable, and 60021 samples; RBuild is data describing residential buildings, which includes 103 features, 1 dependent variable, and 372 samples; CCrime is data describing community crime, which includes 127 features, 1 dependent variable, and 1994 samples. It is worth noting that the UCI datasets obtained from the UCI Machine Learning Repository generally have more missing values; therefore, the mean filling method is used for data processing during the experiment. In this paper, using BlogData, RBuild, and CCrime of the UCI dataset is to compare the regression effects of the new model on the public dataset to verify the reliability and generalization of the new model in our experiments.

Both WYHXB and NYWZ are the basic experimental data of Shenfu injection in the treatment of cardiogenic shock. The experimenters used the left anterior descending coronary artery near the cardiac tip to replicate the metaphase cardiogenic shock rat model and gave the Shenfu injection (unit: ml·kg^−1^) to the shock rat models divided into 7 groups (0.1, 0.33, 1.0, 3.3, 10, 15, and 20, respectively) by the dose of Shenfu injection, in which included 6 rats in each group, and set the model group and blank group in whole experiment meanwhile. After 60 minutes of administration, the pharmacodynamic indicators of the red blood cell flow rate (m/s) were collected. The substance information contained in the Shenfu injection is called exogenous substance (i.e., WYHXB data, as shown in Table 2), and the substance information of the experimental individual itself is called endogenous substance (i.e., NYWZ data, as shown in Table 3). In the two data, the material information is characteristic, and the red blood cell flow rate is the dependent variable.

### 4.2. Results and Discussion

The programming tool used in this experiment is Python 3.6, the operating system is Windows 10, the memory is 8 GB, and the CPU is Intel (R) Core (TM) i5-3230M.

#### 4.2.1. Filtering of Irrelevant Features

In order to ensure the reliability of the new model, the RMSE (root mean square error) of the two regression models of GBDT [30] and XGBoost [31] was adopted as the comprehensive evaluation index, that is, the average value of the two regression models RMSE was taken as the evaluation index, and then the characteristics of the original dataset were filtered by the certain ratio *P* gradually (if the number of features has a decimal number, the result is rounded in the experiment), so that it can be sure that the corresponding RMSE value is the best when what the ratio *P* is taken. And it is more appropriate to judge how many irrelevant features are deleted to achieve the purpose of effectively filtering the irrelevant features, and the experimental results are shown in Table 4.

According to the experimental results in the above Table 4, when *P* = 0.85 in the WYHXB data, the corresponding average RMSE mean value is the best, and 120 irrelevant features are filtered (the original features are 798); when *P* = 0.8 in the NYWZ data, the corresponding average RMSE is the best, and 2057 irrelevant features are filtered (10283 original features); when *P* = 0.5 in the BlogData data, the corresponding RMSE is the best, and 140 irrelevant features are filtered (280 original features); when *P* = 0.7 in RBuild data, its corresponding RMSE mean is the best, and 31 irrelevant features are filtered (103 original features); when CCrime data takes *P* = 0.95, its corresponding RMSE mean is the best, and 7 irrelevant features (127 original features) were filtered. As a result, after filtering the irrelevant features by the above MIC method, a candidate feature subset of five sets of experimental data can be obtained. By further analyzing the candidate feature subsets, it can be found that the RMSE of the original data has little difference with the RMSE of the candidate feature subsets (the experimental results are shown in Table 5); therefore, the features deleted in this experiment have little effect on the accuracy of the model and the process finally filters out irrelevant features and better preserves the features associated with the target variables.

#### 4.2.2. Elimination of Redundant Features

**Figure 2:**
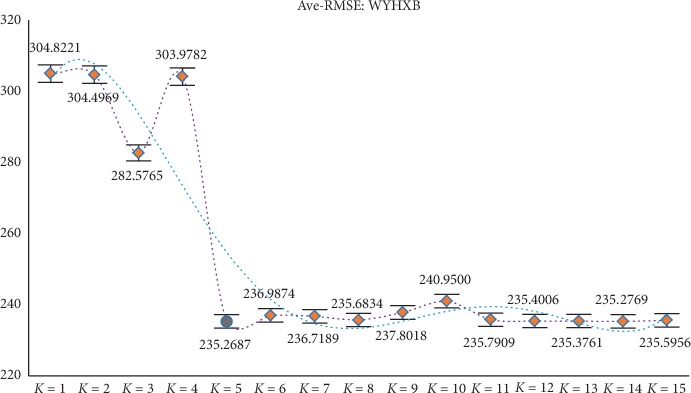
WYHXB parameter *K* selection.

**Figure 3:**
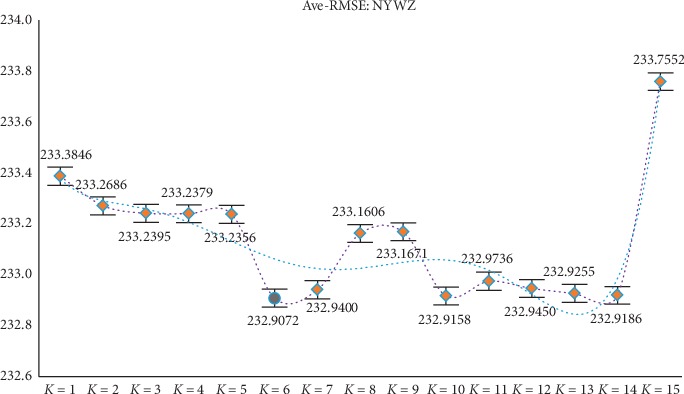
NYWZ parameter *K* selection.

**Figure 4:**
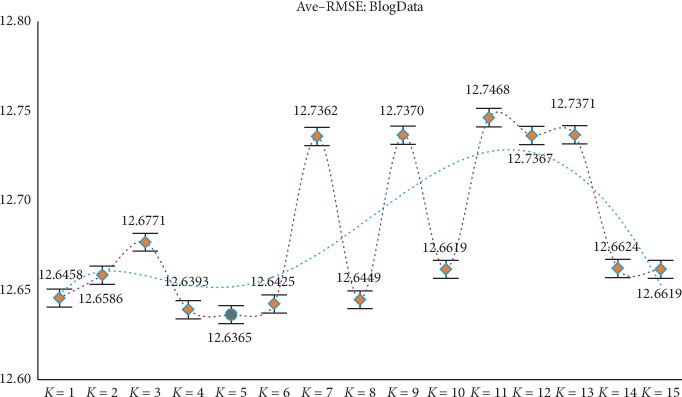
BlogData parameter *K* selection.

**Figure 5:**
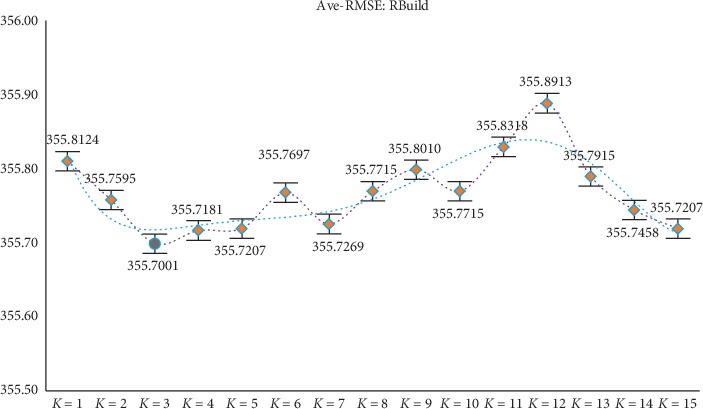
RBuild parameter *K* selection.

**Figure 6:**
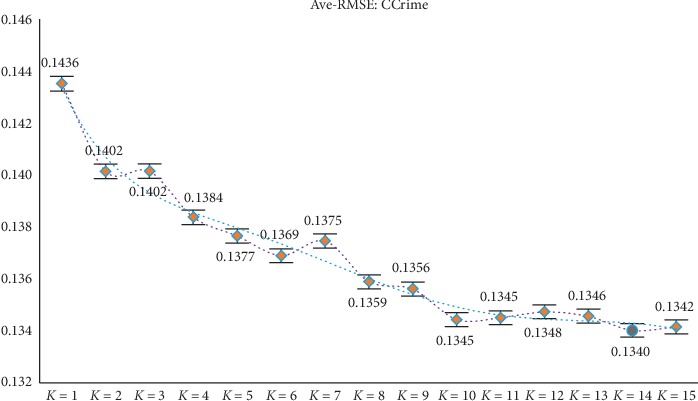
CCrime parameter *K* selection.

Through the above experiments, filtering of irrelevant features can be achieved by obtaining candidate feature subsets. However, according to the construction of the new model, it is necessary to divide the candidate feature subsets (ascending order) equally in the experimental process, but different partitioning strategies will affect the final experimental results, so further discussion and analysis of the parameter *K* are needed (the value range of *K* is set to 1 to 15) to determine the optimal *K* value to ensure the reliability of the model results. At the same time, in order to avoid the contingency of the experiment as much as possible, the experiment still adopts the RMSE of GBDT and XGBoost as the comprehensive evaluation index (i.e., the mean RMSE of the two). After experimental analysis (results shown in Figures 2–6), it can be found that when *k* = 5 of WYHXB data, its corresponding RMSE value is the best; when *k* = 6 of NYWZ data, its corresponding RMSE value is the best; when the *k* = 5 of the BlogData data, the corresponding RMSE value is the best; when the *k* = 3 of the RBuild data, the corresponding RMSE value is the best; when *k* = 14 in the CCrime data, the corresponding RMSE value is the best. After the division of the candidate feature subsets, the redundancy of the features can be analyzed in the later experiments, so as to select the optimal feature subsets.

For further analyzing the model, each dataset was randomly divided into a training set and a test set with the ratio of 6 : 4, and XGBoost [31], Lasso [32], FCBF-MIC [19], and the improved algorithm (CI_AMB) were used for training and learning; the test set was subjected to regression experiment (model parameters selected were consistent with the above experimental results), and RSME was used as the model index. At the same time, in order to ensure the reliability of the model results, each test data was tested 10 times, and then the average value was taken as the final experimental results. In order to verify the effect and effectiveness of the feature selection during the experiment, the original data were also compared using the regression model of GBDT and XGBoost. The experimental results are shown in Tables 6–7:

It can be seen from the experimental results in the above table that the feature selection of the CI_AMB method is performed on the test set of five sets of raw data, and the experimental results are as follows: the number of original features of the WYHXB data is 798, and after the redundancy feature is removed, the final number of optimal feature subsets selected is 80, including 19 strongly correlated features and 61 weakly correlated nonredundant features. The number of original features of NYWZ data is 10283. After the elimination of redundant features, the final number of optimal feature subsets that can be screened is 220, including 59 strongly correlated features and 161 weakly correlated nonredundant features; the original number of BlogData data is 280, after the redundant features are eliminated, and finally, the number of optimal feature subsets that can be screened out is 48, including 5 strongly correlated features and 43 weakly correlated nonredundant features. The number of original features of RBuild data is 103, after the elimination of redundant features. Finally, the number of optimal feature subsets that can be screened out is 35, including 16 strongly correlated features and 19 weakly correlated nonredundant features; the number of original features of CCrime data is 127, after doing the redundancy, and the final number of suboptimal set of features can be selected to 37, including 3 strongly correlated feature and 34 weakly correlated nonredundant feature. It is worth noting that after filtering the irrelevant features and eliminating the redundant features, the obtained strongly correlated features and weakly correlated nonredundant features are distinguished according to the degree of correlation between the features and the target variables, that is, if the MIC score is greater than 0.6, it is a strongly correlated feature, and if not, it is a weakly correlated nonredundant feature.

**Figure 7:**
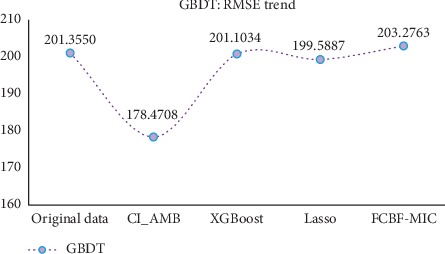
The average RMSE trend of the five sets of datasets.

**Figure 8:**
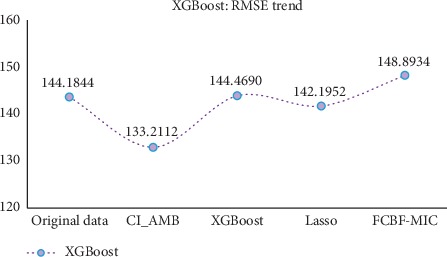
The average RMSE trend of the five sets of datasets.

After the CI_AMB feature selection, it can be found that (1) compared with the original data (in the case of no feature selection), the new method has the slightly inferior result (0.0024 greater error than the result of the original data) in the CCrime data (using the RMSE of GBDT as the evaluation index, Table 6), but in other datasets, the results are better than the original data (see Table 6 and 7); (2) compared with XGBoost, Lasso, and FCBF-MIC, while the number of features is similar, the RMSE values of the evaluation models in the CI_AMB method are better than in other methods. At the same time, in order to observe and compare the experimental results more intuitively, the trend graphs of the two evaluation indicators (GBDT and XGBoost) were plotted (Figures 7 and 8) to reflect the overall fluctuation of the RMSE. Combining the above table with the experimental results of Figures 7 and 8, it can be observed that the improved algorithm is generally superior to other algorithms, indicating that the new model is effective in removing the effects of irrelevant features and redundant features. In summary, not only can the improved algorithm better filter out the strongly correlated features and weakly correlated nonredundant features, but also improves the regression accuracy of the model to some extent.

## 5. Conclusions

Aiming at the problem that the basic experimental data of TCM present high dimensionality and few samples and contain more irrelevant information and redundant information, a hybrid feature selection method based on iterative approximation Markov blanket is proposed. The method performs two-stage feature analysis by the maximum information coefficient and iterative approximation Markov blanket, respectively, to do filtering of unrelated features and culling of redundant features, so as to achieve the screening of optimal feature subsets. Through the experimental comparison between the basic data of Chinese medicine and UCI dataset, it is proved that the improved algorithm significantly reduces the feature dimension and improves the interpretation degree of the model. It is a kind of analysis suitable method for high-dimensional small sample data of traditional Chinese medicine. In the next research work, we will continue to optimize the algorithm and ensure the reasonable setting of relevant parameters can be further studied when building the model.

## Figures and Tables

**Algorithm 1:**
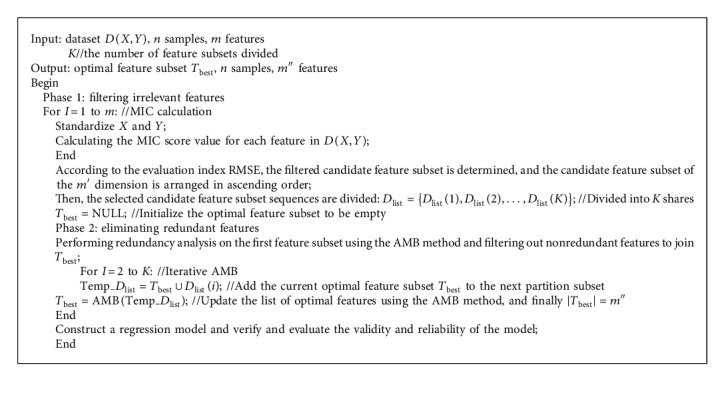
CI_AMB algorithm.

**Table 1:** Basic dataset information (default task: regression).

Datasets	Number of samples	Number of attributes
WYHXB	54	799 (798 + 1)
NYWZ	54	10284 (10283 + 1)
BlogData	60021	281 (280 + 1)
RBuild	372	104 (103 + 1)
CCrime	1994	128 (127 + 1)

**Table 2:** Partial data of basic experiments with traditional Chinese medicine substances (WYHXB).

0.34_237.0119 *m*/*z*	0.35_735.1196 *m*/*z*	0.36_588.0942 *m*/*z*	…	0.36_590.0903 *m*/*z*	Red blood cell flow rate (*μ*·m/s)
0.48808	302.16	0	…	27.8589	750
100.078	62.016	0	…	3.80712	1400
11.6992	52.5058	7.61005	…	4.85059	785
143.643	284.113	0	…	456.607	790
7.75089	54.4535	0	…	0	670
18.2499	0	0	…	14.6621	680
…	…	…	…	…	…
28.5783	0	0	…	2.3551	850
2.91064	0	16.1624	…	3.41406	620
…	…	…	…	…	…

**Table 3:** Partial data of basic experiments with traditional Chinese medicine substances (NYWZ).

11.10_787.5077 *m*/*z*	12.29_526.1784 *m*/*z*	12.29_531.2005 *m*/*z*	…	12.47_631.3847 *m*/*z*	Red blood cell flow rate (*μ*·m/s)
53.3719	11557.6	764.329	…	1795.79	2200
43.4717	7971.33	875.465	…	1842.39	2750
76.507	3399.9	870.161	…	1562.81	1980
153.145	51027.4	916.064	…	1619.62	1860
16.3197	10694.4	942.699	…	1612.42	2100
42.2836	11048.1	714.536	…	1649.23	2000
…	…	…	…	…	…
55.5021	4702.83	748.844	…	1632.9	2481
153.21	78912.8	835.24	…	1647.55	2970
…	…	…	…	…	…

**Table 4:** Experimental results of five datasets of filter-independent features.

*P*	WYHXB		NYWZ		BlogData		RBuild		CCrime	
	Number of features	Ave-RMSE	Number of features	Ave-RMSE	Number of features	Ave-RMSE	Number of features	Ave-RMSE	Number of features	Ave-RMSE
**0.95**	758	234.960328	9768	233.324863	266	12.645784	97	354.101779	**120**	**0.131535**
0.9	718	235.019819	9254	233.324863	252	12.645784	92	354.090179	114	0.131695
**0.85**	**678**	**234.800187**	8740	233.324863	238	12.645784	87	354.134252	107	0.131858
**0.8**	638	235.133101	**8226**	**233.324863**	224	12.645784	82	354.146541	101	0.131792
0.75	598	235.104648	7712	233.388367	210	12.645784	77	353.914801	95	0.131897
**0.7**	558	235.132128	7198	233.388367	196	12.645784	**72**	**353.914801**	88	0.131853
0.65	518	235.191663	6683	233.385479	182	12.645784	66	353.923275	82	0.131902
0.6	478	235.202756	6169	233.394604	168	12.645784	61	354.042364	76	0.132113
0.55	438	235.263138	5655	233.394604	154	12.645784	56	354.050328	69	0.132164
**0.5**	399	235.962421	5141	233.357302	**140**	**12.645784**	51	354.053246	63	0.132310
0.45	359	235.941428	4627	233.355757	126	12.649723	46	354.770411	57	0.132497
0.4	319	236.399412	4113	233.354086	112	12.651157	41	354.849084	50	0.132620
0.35	279	236.574098	3599	233.354248	98	12.657242	36	355.659524	44	0.133428
0.3	239	376.546789	3084	233.358374	84	12.664293	30	355.714190	38	0.133759
0.25	199	406.768586	2570	233.399275	70	12.671595	25	355.700106	31	0.134865
0.2	159	445.621765	2056	233.437486	56	12.676944	20	355.714027	25	0.136386
0.15	119	545.521345	1542	233.539485	42	12.677181	15	355.722452	19	0.137433
0.1	79	553.326100	1028	233.550540	28	12.677343	10	355.785519	12	0.139937

**Table 5:** Comparison of experimental data between raw data and candidate feature subsets.

	Original data		Candidate feature subset	
	Number of features	RMSE	Number of features	RMSE
WYHXB	798	234.967849	678	234.800187
NYWZ	10283	234.052699	8226	233.324863
BlogData	280	12.645784	140	12.645784
RBuild	103	352.473674	72	353.914801
CCrime	128	0.131377	120	0.131535

**Table 6:** Comparison of experimental results between CI_AMB and other methods (RMSE evaluation index of GBDT).

	Original data		CI_AMB		XGBoost		Lasso		FCBF-MIC	
	Number of features	RMSE	Number of features	RMSE	Number of features	RMSE	Number of features	RMSE	Number of features	RMSE
WYHXB	798	267.5115	80(19 + 61)	**232.7352**	83	269.1644	89	255.9661	15	265.0474
NYWZ	10283	258.4021	220(59 + 161)	**234.8831**	212	263.3908	215	256.2172	60	265.2352
BlogData	280	22.7247	48(5 + 43)	**7.4822**	43	14.5660	47	18.7933	9	24.2629
RBuild	103	458.0302	35(16 + 19)	**417.1441**	23	458.2780	26	466.8546	3	461.7130
CCrime	127	**0.1067**	37(3 + 34)	0.1091	37	0.1176	31	0.1121	5	0.1231
Average value		201.3550		**178.4708**		201.1034		199.5887		203.2763

**Table 7:** Comparison of experimental results of CI_AMB with other methods (RMSE evaluation index of XGBoost).

	Original data		CI_AMB		XGBoost		Lasso		FCBF-MIC	
	Number of features	RMSE	Number of features	RMSE	Number of features	RMSE	Number of features	RMSE	Number of features	RMSE
WYHXB	798	227.9061	80(19 + 61)	**205.0669**	83	221.8774	89	214.0560	15	229.7367
NYWZ	10283	219.7160	220(59 + 161)	**201.5748**	212	220.3312	215	225.1712	60	225.1525
BlogData	280	8.6356	48(5 + 43)	**4.1587**	43	10.0949	47	10.2909	9	10.8045
RBuild	103	264.5195	35(16 + 19)	**255.1114**	23	269.8928	26	261.3095	3	278.6242
CCrime	127	0.1447	37(3 + 34)	**0.1443**	37	0.1487	31	0.1483	5	0.1492
Average value		144.1844		**133.2112**		144.4690		142.1952		148.8934

## Data Availability

The traditional Chinese medicine data used in this study can be obtained by contacting the first author. The UCI datasets can be obtained through the UCI Machine Learning Repository (http://archive.ics.uci.edu/ml/datasets.html). It should be noted that the UCI datasets are commonly used standard test datasets proposed by the University of California, Irvine, for machine learning.
